# Understanding Broad Consent

**DOI:** 10.31486/toj.19.0088

**Published:** 2020

**Authors:** John W. Maloy, Pat F. Bass

**Affiliations:** ^1^ Assistant Vice Chancellor for Research Management, Louisiana State University Health Sciences Center Shreveport, Shreveport, LA; ^2^ Office of Research, Louisiana State University Health Sciences Center Shreveport, Shreveport, LA

**Keywords:** *Ethics committees–research*, *informed consent*, *research*, *research personnel*, *research subjects*

## Abstract

**Background:** The 2018 revisions to the Common Rule that were effective in January 2019 introduced a new category of informed consent: broad consent.

**Methods:** Investigators and institutional review board (IRB) members need to understand (1) what broad consent is, (2) the role of broad consent under the revised Common Rule, (3) how and when broad consent can be used, (4) exempt research categories that relate to broad consent, and (5) the scope of limited IRB review as it relates to broad consent.

**Results:** Under the prior regulations, researchers had two consent options: obtain study-specific informed consent or request the IRB to waive the requirement to obtain informed consent. The revision to the Common Rule introduced the third option of broad consent, but its applicability is limited. Broad consent can only be used to obtain an individual's consent for the storage, maintenance, and secondary research use of identifiable private information or identifiable biospecimens. The regulatory authority for broad consent is at 45 CFR §46.116(d). None of the required elements of broad consent can be omitted or altered because each element is considered essential. Broad consent shares many of the requirements for study-specific informed consent, but several elements are unique: a description of the types of secondary research that may be conducted; statements describing the private information or biospecimens that might be used in research, whether sharing of the information or biospecimens might occur, and the types of institutions or researchers that might conduct research with the information or biospecimens; information on how long the information or biospecimens may be stored, maintained, and used; a statement that subjects will or will not be informed of the details of any subsequent research; a statement that research results will or will not be disclosed to subjects; and contact information for obtaining answers to questions about the subjects’ rights regarding storage and use of information or biospecimens and whom to contact regarding research-related harm.

**Conclusion:** Broad consent provides flexibility that did not exist prior to the revision, giving researchers the option to obtain broad consent for the storage, maintenance, and secondary research use of identifiable private information or identifiable biospecimens. With an understanding of the regulations, an investigator can plan how best to organize his or her research plan and decide whether to obtain study-specific informed consent, to apply for a waiver of consent, or to obtain broad consent.

## INTRODUCTION

The Federal Policy for the Protection of Human Subjects—the Common Rule—is the set of regulations governing human subjects research that has been adopted by various federal departments and agencies.^1^ Each federal department or agency that follows the Common Rule has adopted regulations that are substantively identical to the Department of Health and Human Services regulations at 45 CFR §46, Subpart A.^1^ The regulations specify the type of information that must be given to a potential research subject before he or she agrees to participate in research and who may give permission to participate; provide protections for vulnerable populations such as children, prisoners, and the mentally disabled; and address issues of research subject injury.

A committee called the institutional review board (IRB) has the primary responsibility for protecting the rights and welfare of people who participate in research, and the Common Rule provides the regulatory framework within which the IRB performs the function of ensuring that research involving human subjects is conducted ethically.

A major revision to the Common Rule was published in the Federal Register on January 19, 2017.^2^ The revision had an effective date of July 19, 2018 and a general compliance date of January 21, 2019.^3^ One of the changes was the introduction of the concept of broad consent. To help researchers and IRB members understand the issues related to broad consent, this article (1) defines broad consent and discusses its elements, (2) examines the role of broad consent under the revised Common Rule, (3) explains when broad consent can be used, and (4) discusses exempt research categories related to broad consent and the limited IRB reviews associated with the exemptions.

## DEFINITION AND ELEMENTS OF BROAD CONSENT

Prior to its revision, the Common Rule regulated informed consent, and the Common Rule continues to do so after the revision. Informed consent is the process “whereby individuals are to be fully informed, in understandable language, about all aspects of research studies to enable informed decision making [about whether to participate in a research study].”^4^ Under the prior regulations, researchers had two consent options: obtain study-specific informed consent or request the IRB to waive the requirement to obtain informed consent. The revision to the Common Rule introduced a third option: broad consent. Broad consent can be obtained instead of informed consent but *only* with respect to the storage, maintenance, and secondary research use of identifiable private information or identifiable biospecimens.^5^ Broad consent is not a waiver but an alternative to study-specific consent.

Key terms are *secondary research* and *identifiable private information or biospecimens*. Secondary research refers to research with materials originally obtained for nonresearch purposes or for research other than a current research study.^5^ Identifiable private information is information about which the subject has a reasonable expectation of privacy but from which the subject's identity can be determined, such as a medical record. An identifiable biospecimen is a biospecimen from which the identity of the subject can be determined, such as a blood or tissue sample labeled with information that identifies the individual.

The regulatory authority for broad consent is at 45 CFR §46.116(d).^6^ No templates for broad consent forms were provided in the rule, so institutions can develop their own forms to satisfy the requirements. However, none of the elements specified in the rule can be omitted or altered because each element is considered essential.

Broad consent requires most of the general requirements of study-specific informed consent:

1. Obtaining the legally effective informed consent of the subject or the subject's legally authorized representative.
2. Seeking informed consent under circumstances that provide an opportunity to discuss and consider whether or not to participate and that minimize the possibility of coercion or undue influence.
3. Providing information in understandable language.
4. Providing information that a reasonable person would want to have to make an informed decision about whether to participate and providing an opportunity to discuss that information.
5. Avoiding exculpatory language. Exculpatory language either waives or appears to waive the subject's legal rights, or it releases or appears to release the investigator, the sponsor, the institution, or its agents from liability for negligence.^6^

Broad consent also requires four of the basic elements of study-specific informed consent:

1. A description of any reasonably foreseeable risks or discomforts to the subject.
2. A description of any benefits to the subject or to others that may reasonably be expected from the research.
3. A statement describing the extent, if any, to which confidentiality of records identifying the subject will be maintained.
4. A statement that participation is voluntary and that the subject may choose not to participate or discontinue participation at any time without penalty or loss of benefits to which the subject is otherwise entitled.^6^

Two additional elements must be included in broad consent when appropriate: a statement that the subject's biospecimens—even if identifiers are removed—may be used for commercial profit and whether the subject will or will not share in the profit and a statement indicating if the research will or might include whole genome sequencing.^6^

### Unique Elements

A number of elements are unique to broad consent.^6^ First, the consent must include a general description of the types of research that may be conducted, and the information must be sufficient for a reasonable person to conclude that he or she would consent to the types of research anticipated. Identifying every conceivable type of research that might be conducted is neither possible nor desirable, so broad categories of descriptions should satisfy the regulatory requirement. However, if possible future research could raise particularly sensitive ethical, moral, religious, or cultural issues—for example, genetic research or controversial research such as research using embryonic stem cells—respect for the person of a research participant suggests erring on the side of disclosing that type of research in the description.

Second, the consent must include statements describing the identifiable private information or identifiable biospecimens that might be used in research, whether sharing of the information or biospecimens might occur, and the types of institutions or researchers that might conduct research with the information or biospecimens.

Third, the consent must describe how long the information or biospecimens may be stored and maintained and how long the information or biospecimens may be used for research purposes. These time periods may be indefinite.

Fourth, if the subject or his or her legally authorized representative will not be provided details about specific research studies that might be conducted using the information or biospecimens, a statement must be included that advises the subject or his or her legally authorized representative of this fact and of the possibility that he or she might have chosen not to consent to some of those specific research studies.

Fifth, the consent must include a statement that clinically relevant research results may not be disclosed to the subject.

Finally, information must be provided regarding whom to contact for answers to questions about the subject's rights regarding storage and use of information or biospecimens and whom to contact regarding research-related harm.

## THE ROLE OF BROAD CONSENT UNDER THE REVISED COMMON RULE

As stated earlier, prior to the revision of the Common Rule, broad consent did not exist. Researchers now have the option to obtain broad consent for the storage, maintenance, and secondary research use of identifiable private information or identifiable biospecimens.^7^ The broad consent option provides flexibility that did not exist prior to the revision.

Under the prior regulations, secondary research with identifiable private information or identifiable biospecimens required either that the IRB waive the requirement to obtain informed consent or that study-specific informed consent be obtained. To grant a waiver of consent, the IRB had to determine and document that (1) the research involved no more than minimal risk; (2) the waiver would not adversely affect the rights and welfare of the subjects; (3) the research could not practicably be carried out without the waiver; and (4) whenever appropriate, the subjects had to be provided with additional pertinent information after participation.

The revision to the Common Rule retained these requirements with minor modifications.^6^ The revision added the requirement that the IRB determine and document that the research using identifiable private information or identifiable biospecimens could not practicably be carried out without using the information or biospecimens in an identifiable format. In addition, the revision added “legally authorized representatives” to requirement number 4.

## USING BROAD CONSENT

The use of broad consent is not mandatory. Researchers may continue to obtain a waiver of consent, continue to obtain study-specific consent, or use the broad consent option. Table 1 provides sample scenarios addressing the choice to use broad consent, study-specific consent, or a waiver of consent. The decision should rest with the principal investigator who must determine which procedure works best for the research project.

**Table 1.** Scenarios Addressing the Choice to Use Broad Consent, Study-Specific Consent, or Waiver of Consent

**Background:** An AMC has a National Cancer Institute designation. The AMC has a cancer research center that not only conducts primary research but also operates a tissue bank that regularly supplies tissue samples to investigators at the AMC and to investigators at other institutions. A physician at the AMC both treats patients and conducts cancer-related clinical trials. The representative of a large pharmaceutical company has approached him about participating in a multisite clinical trial of a new drug to treat prostate cancer. The physician believes he has several patients who would benefit from participation in the study. The pharmaceutical company is especially interested in recruiting minority research subjects. The recruitment goal for each site is 7 to 10 subjects. After recruitment into the study, a biopsy will be performed, and the tumor will be given a Gleason score. The subject will be administered the test drug for 6 months, and additional biopsies will be taken in the 6th month and the 12th month of the study. The results of the biopsies will be compared to determine the efficacy of the drug. The samples will be labeled with the subject's name, age, race, and sex. The physician has requested and obtained permission to collect additional tissue samples to be placed in the AMC's tissue bank. The company would like to start the trial as quickly as possible.	
Drug study consent	For the study sponsored by the pharmaceutical company, the physician must obtain study-specific consent. Because a test drug and prostate biopsy present greater than minimal risk, he cannot obtain a waiver of consent from the IRB. Further, because the research subjects are his patients, the physician cannot argue that the research could not practically be carried out without the waiver. Although the biopsied tissue is identifiable, he cannot use broad consent because this study is not secondary research. His only option is to obtain a study-specific consent to conduct the drug study.
Tissue sample collection consent	With regard to the collection of additional tissue samples to be stored in the AMC's tissue bank, the physician could obtain broad consent now to collect and store the tissue for use in research at some future date. The tissue samples are identifiable and would be collected for secondary research, not the current study.
Secondary research consent	If the physician decides to conduct research with the stored tissue, he has the widest range of options. If he wants to perform genetic tests on samples obtained from the tissue bank, he could seek study-specific consent from the original donors to conduct the genetic testing. If the criteria for a waiver of consent are met, he could obtain a waiver of consent from the IRB for the genetic testing. If broad consent had been obtained at the time of the drug study and if the scope of the broad consent were sufficiently broad to cover genetic testing, the physician could conduct the study without having to obtain additional consent from the donor.

AMC, academic medical center; IRB, institutional review board.

Certain types of research projects require study-specific informed consent. Study-specific consents provide detailed information about the particular study (rather than the broad consent form's general description of the types of research that might be conducted), allow for broad IRB review of informed consent, and allow for approval of more types of research than either broad consent or a waiver does. With study-specific informed consent, the subject agrees to participate in a single research study that may involve a particular drug, device, or disease process. If the subject wants to participate in a different study, he or she must be consented again for the new study.

However, trying to obtain informed consent is sometimes impractical. For example, if someone submits a biospecimen to a biobank or other depository, and several years later, an investigator wants to use that biospecimen in a research study, the donor may have moved or his or her contact information may have been lost. In cases such as these, a waiver of consent from the IRB would allow the research to be performed even though the subject did not provide informed consent.

Broad consent is an alternative to study-specific consent, not a waiver of consent. Broad consent can be a useful option because it allows an investigator to obtain data or biospecimens in association with a current study that can be used in future research without having to re-consent the research participant. In general, broad consent must be obtained before the storage, maintenance, or secondary research use of identifiable private information or identifiable biospecimens occurs. Obtaining such consent commonly occurs when blood or tissue samples are collected for storage in a biorepository. If the same or another investigator decides to conduct secondary research using the identifiable information or biospecimens at a later date, the investigator would not need to obtain additional consent if the scope of the original broad consent was sufficient to cover the proposed research study.

What happens if the original broad consent did not sufficiently cover the use of identifiable information or identifiable biospecimens in the currently proposed research study? What happens if no consent to conduct secondary research was obtained when the identifiable private information or identifiable biospecimens were collected? In both scenarios, the answer depends on the sample size of the information or biospecimens and on the investigator's objective. If the sample size is small and if the investigator is only interested in conducting the currently proposed study, obtaining study-specific informed consent may be the best option. If the sample size is small but the investigator is not sure if the information or biospecimens will need to be used again, obtaining a new broad consent is the best option. If the new broad consent is drafted properly, the investigator would not only obtain consent to use the identifiable private information or identifiable biospecimens in the currently proposed study but would also secure use of the information or biospecimens for later studies. If the sample size is large but the study is important, a waiver of consent may be the best option. Among other requirements, the investigator would have to prove to the IRB that the research could not practicably be carried out without the waiver. However, if an initial request for broad consent was made but the research participant declined, the IRB could not grant a waiver.

### Broad Consent Limitations

Broad consent is subject to several limitations. Table 2 provides sample scenarios illustrating these limitations.

**Table 2.** Scenarios Illustrating Limitations to the Use of Broad Consent

Study/Specimen Type	Research Scenario
Primary research	An investigator plans to participate in a phase 2 study of a pediatric asthma drug. The investigator cannot use broad consent for this study because the asthma drug study is primary research; the investigator will be performing an intervention (ie, administering the study drug). Broad consent is only available for secondary research.
Secondary research/Identifiable specimens	A physician is collecting blood samples from research participants in a diabetes study. From experience, she has learned how difficult it is to re-consent research participants. She would like to collect blood samples for additional research in the future and label the samples with private health information related to the research participant. The physician can use broad consent in this context. Because of the inclusion of private health information, the samples will be identifiable, and the samples will be used in secondary research (the physician will not be using the samples in her current diabetes study).
Secondary research/Identifiable specimens	An investigator seeks broad consent from a potential research subject to obtain tissue samples for a university's tissue bank to be used in secondary research at a later time. During the consenting process, the subject asks about the types of research that might be done with the tissue samples. The subject wants an assurance that the tissue will not be used for certain types of research. The investigator explains the general types of research that might be performed and mentions research that the subject finds objectionable. The subject declines to sign the broad consent. The investigator cannot use this patient's tissue samples. The IRB cannot grant a waiver of consent once the investigator sought broad consent and the subject declined to grant it.
Secondary research/Deidentified specimens	An investigator wants to conduct research on deidentified tissue samples from the institution's tissue bank. Broad consent is not an option, because broad consent only applies if the samples are identifiable. Because the tissue samples are deidentified, obtaining study-specific informed consent may not be possible. Therefore, the investigator should seek a waiver of consent from the IRB.
Specimens collected for nonresearch purposes	In addition to her medical practice, a physician also conducts clinical research. A patient schedules a visit for a yearly wellness examination. As part of the examination, routine blood work is done; more blood was collected than necessary to complete these tests. The physician is also conducting a diabetes study comparing A1c levels of different ethnic groups. She wants to use broad consent to obtain the patient's permission to use the extra blood in her diabetes study; however, she cannot use broad consent because the blood was collected for nonresearch purposes. In this case, the detection of clinical abnormalities was part of a preventive treatment regime.

IRB, institutional review board.

First, broad consent cannot be used for primary research. Primary research involves an interaction or intervention with the research subject. Interactions include communication or interpersonal contact between the investigator and the subject.^7^ An interview is an interaction. An intervention is a procedure in which information or biospecimens are gathered^7^; an example is drawing a blood sample. Manipulations of the subject or the subject's environment that are performed for research purposes are also interventions.^7^ For example, an investigator might alter the temperature in a classroom to study the effect on concentration.

Second, the use of broad consent is strictly limited to secondary research.^2^ As stated earlier, secondary research is research conducted using data or specimens that were collected or obtained in prior research. For example, if an investigator developed an exercise program for patients with diabetes, collected quality of life data as part of the exercise study, and later used the quality of life data in another study comparing life quality among age groups, the latter study is secondary research.

Third, broad consent cannot be used for materials collected for nonresearch purposes. An example is specimens that are left over from routine clinical diagnosis or treatment.

Fourth, broad consent is limited to research involving identifiable private information or identifiable biospecimens.^2^

Fifth, broad consent is not available for research that is not human subjects research. For example, a study using data from the Bureau of Justice Statistics to determine whether a particular demographic group is more likely to commit a hate crime is not human subjects research. No consent would be required in this case because the collection and use of the data, including for research purposes, are authorized by statute.^8^ In contrast, a biography of an individual is generally not human subjects research because it does not contribute to generalizable knowledge,^7^ but some sources of information used to write the biography may require the subject's consent. For example, if the biographical subject served in the military, and <62 years have passed since the person's date of discharge, the only information that can be obtained from the subject's Official Military Personnel File without the person's consent or the consent of the next of kin is name, past and present positions, titles, salaries, grades, and job locations.

Finally, broad consent gives subjects the option to decline storage, maintenance, and secondary research use of their data or biospecimens. If a research subject declines to give broad consent, the IRB cannot subsequently grant a waiver of consent.^9^

## EXEMPT RESEARCH AND LIMITED INSTITUTIONAL REVIEW BOARD REVIEW RELATED TO BROAD CONSENT

The revision of the Common Rule modified and amended the categories of research that are exempt from compliance with the Common Rule. There are now eight such exemptions; two of these exemptions relate to broad consent. These exemptions provide a pathway for obtaining consent for the use of identifiable private information and identifiable biospecimens that did not exist before the revision of the Common Rule.

The proper use of broad consent can make the storage, maintenance, and secondary research of identifiable information or biospecimens exempt from compliance with the Common Rule but not exempt from IRB oversight. Both exemptions require limited IRB review. As part of these limited reviews, privacy and confidentiality are reviewed in the same way as for nonexempt human subjects research.^2^ The expedited review process, in which the chair of the IRB or an experienced IRB member designated by the chair conducts the review, may be used.^10^ Alternatively, the convened IRB may conduct the review.

### Storage or Maintenance Exemption

The first exemption states “Storage or maintenance for secondary research for which broad consent is required: Storage or maintenance of identifiable private information or identifiable biospecimens for potential secondary research use if an IRB conducts a limited IRB review and makes the determinations required by §46.111(a)(8).”^11^ According to 45 CFR §46.111(a)(8),^12^ the IRB is required to make three specific determinations: (1) the broad consent complies with the requirements of §46.116,^6^ (2) the broad consent is appropriately documented, and (3) the research plan contains adequate provisions to protect subject privacy and maintain confidentiality of the data if a change is made to how the identifiable information or biospecimens are stored or maintained.

### Secondary Research Exemption

The second exemption is for secondary research, and the rule specifies the required IRB findings.^11^ Secondary research involving the use of identifiable private information or identifiable biospecimens is exempt if the IRB determines that following criteria were met: (1) broad consent was obtained and documented, (2) the research is within the scope of the broad consent, and (3) the investigator does not include returning individual research results to subjects as part of the study plan.

## CONCLUSION

Institutions that have the resources and willingness to provide broad consent as an option to investigators must understand the mechanisms of its use. These mechanisms include the role of broad consent under the revised Common Rule, when broad consent may be appropriately used, exemptions from compliance with the Common Rule that may be applicable, and the scope of the IRB review.

## Notes

This article meets the Accreditation Council for Graduate Medical Education and the American Board of Medical Specialties Maintenance of Certification competencies for Patient Care, Medical Knowledge, and Systems-Based Practice.
